# C-Myc protein expression indicates unfavorable clinical outcome in surgically resected small cell lung cancer

**DOI:** 10.1186/s12957-024-03315-7

**Published:** 2024-02-19

**Authors:** Christian Lang, Zsolt Megyesfalvi, Andras Lantos, Felicitas Oberndorfer, Mir Alireza Hoda, Anna Solta, Bence Ferencz, Janos Fillinger, Anna Solyom-Tisza, Alessandro Saeed Querner, Felix Egger, Kristiina Boettiger, Thomas Klikovits, Gerald Timelthaler, Ferenc Renyi-Vamos, Clemens Aigner, Konrad Hoetzenecker, Viktoria Laszlo, Karin Schelch, Balazs Dome

**Affiliations:** 1 Department of Thoracic Surgery; Comprehensive Cancer Center, Medical University of Vienna, Waehringer Guertel 18-20, Vienna, A-1090 Austria; 2 Department of Medicine II, Division of Pulmonology, Medical University of Vienna, Vienna, Austria; 3 National Korányi Institute of Pulmonology, Budapest, Hungary; 4 Department of Thoracic Surgery, National Institute of Oncology-Semmelweis University, Ráth György u. 7-9, Budapest, 1122 Hungary; 5 Department of Pathology, Medical University of Vienna, Vienna, Austria; 6 Department of Thoracic Surgery, Clinic Floridsdorf, Vienna, Austria; 7 Center for Cancer Research, Medical University of Vienna, Vienna, Austria; 8 National Institute of Oncology and National Tumor Biology Laboratory, Budapest, Hungary; 9 Department of Translational Medicine, Lund University, Lund, Sweden

**Keywords:** Small cell lung cancer, Myc family, Molecular subtypes, Immunohistochemistry

## Abstract

**Background:**

By being highly involved in the tumor evolution and disease progression of small cell lung cancer (SCLC), Myc family members (C-Myc, L-Myc, and N-Myc) might represent promising targetable molecules. Our aim was to investigate the expression pattern and prognostic relevance of these oncogenic proteins in an international cohort of surgically resected SCLC tumors.

**Methods:**

Clinicopathological data and surgically resected tissue specimens from 104 SCLC patients were collected from two collaborating European institutes. Tissue sections were stained by immunohistochemistry (IHC) for all three Myc family members and the recently introduced SCLC molecular subtype-markers (ASCL1, NEUROD1, POU2F3, and YAP1).

**Results:**

IHC analysis showed C-Myc, L-Myc, and N-Myc positivity in 48%, 63%, and 9% of the specimens, respectively. N-Myc positivity significantly correlated with the POU2F3-defined molecular subtype (*r* = 0.6913, *p* = 0.0056). SCLC patients with C-Myc positive tumors exhibited significantly worse overall survival (OS) (20 vs. 44 months compared to those with C-Myc negative tumors, *p* = 0.0176). Ultimately, in a multivariate risk model adjusted for clinicopathological and treatment confounders, positive C-Myc expression was confirmed as an independent prognosticator of impaired OS (HR 1.811, CI 95% 1.054–3.113, *p* = 0.032).

**Conclusions:**

Our study provides insights into the clinical aspects of Myc family members in surgically resected SCLC tumors. Notably, besides showing that positivity of Myc family members varies across the patients, we also reveal that C-Myc protein expression independently correlates with worse survival outcomes. Further studies are warranted to investigate the role of Myc family members as potential prognostic and predictive markers in this hard-to-treat disease.

**Supplementary Information:**

The online version contains supplementary material available at 10.1186/s12957-024-03315-7.

## Introduction

Small cell lung cancer, one of the most aggressive types of lung cancer, is characterized by a rapid doubling time, strong predilection for early metastasis and dismal prognosis [1]. Worldwide, more than 300,000 new small cell lung cancer (SCLC) cases are being detected [2]. Despite all efforts to improve the clinical outcomes, the prognosis of SCLC patients did not change significantly over the past decades and the 5-year survival rates are still way below 10% [3]. Accordingly, new and more effective therapeutic strategies are clearly needed for this devastating disease.

Although SCLC has long been regarded both clinically and biologically as a homogenous entity, recent evidence arising from preclinical and clinical profiling studies indicates that SCLCs can be divided into distinct molecular subtypes. This novel subtype classification is primarily based on the relative expression of four key transcriptional factors: ASCL1 (SCLC-A subtype), NEUROD1 (SCLC-N), POU2F3 (SCLC-P), and YAP1 (SCLC-Y) [4]. Of note, beyond their prognostic significance, these major SCLC subtypes have distinct neuroendocrine profiles and unique therapeutic vulnerabilities [4–7]. Moreover, it has been recently indicated that the presence of these molecular subtypes is greatly influenced by strong intra-tumoral heterogeneity and that a temporal plasticity between the subtypes might also exists [5]. Importantly, we are only at the beginning to understand whether tumor evolution between subtypes reflects lineage plasticity or differential selection among pre-existing subclones due therapy. Nevertheless, it is anticipated that besides the cytotoxic effects of chemotherapy, Myc oncogene family members also contribute to this dynamic progress of subtype switching [5, 8, 9].

The Myc family consists of three distinct members (MYC, MYCL, and MYCN), each of them coding for three distinct transcription factors (C-Myc, L-Myc, and N-Myc). As for its functions, Myc regulates a wide range of physiological cellular processes such as cell cycle, growth, or metabolism and strongly contributes to the progression of most human tumors including SCLC [10, 11]. Notably, Myc family members have been previously demonstrated to be critical factors in SCLC initiation and progression both in vitro* and *in vivo [9]. Moreover, they represent potent regulators of several clinically relevant processes, such as the development of chemoresistance, or the temporal evolution of SCLC molecular subtypes [8, 12, 13].

Interestingly, increased Myc expression has been demonstrated to associate with poor prognosis in various malignancies [14–16]. While targeting oncogenic Myc has been considered a promising novel therapeutic strategy for malignancies, finding effective ways for its inhibition has remained a challenge for decades [17]. Fortunately, recent efforts have resulted in the development of highly effective direct Myc inhibitors and initial clinical trials have already started to evaluate the feasibility of certain Myc inhibitors in patients with various malignancies [18, 19]. Importantly, there is a clear rationale for targeting Myc in SCLC as well, and the first preclinical results concerning the efficacy of Myc-inhibition are indeed promising [20, 21]. Here, we evaluated the expression profile and clinical relevance of Myc, as well as its co-expression pattern with the subtype-defining transcription factors in a cohort of surgically-treated Caucasian SCLC.

## Materials and methods

### Patients and study design

In this multi-institutional retrospective study, formalin-fixed paraffin-embedded (FFPE) SCLC blocks and corresponding clinicopathological data were collected from patients undergoing surgery between January 2000 and December 2019 at the Medical University of Vienna (Austria) or in the National Koranyi Institute of Pulmonology (Hungary). Clinical follow-up and survival data were retrospectively extracted from institutional medical records and/or archives of the national statistical offices. Notably, all patients have been clinically managed based on the recommendations of a multidisciplinary tumor-board consisting of board-certified pulmonary-, radiation-, and surgical-oncologists in accordance with the recommendations of the contemporary National Comprehensive Cancer Network (NCCN) guidelines [22]. In particular, routine preoperative staging included the following conventional radiological examinations: whole-body computed tomography scan (CT), CT or magnetic-resonance-imaging (MRI) scan of the brain, whole-body fluorodeoxyglucose (FDG)-positron emission tomography (PET), or whole-body skeletal scintigraphy. Adjuvant therapy was defined as when at least 4 cycles of adjuvant chemotherapy have been conducted either in combination with adjuvant irradiation or without adjuvant irradiation. Chemotherapy regimens comprised platinum-based agents (cisplatin or carboplatin) in combination with the topoisomerase-inhibitor etoposide. Routine oncological follow-up was carried out for all patients, comprising of regular (i.e., every 3 months in the first postoperative year and then every 6–12 months) appointments in an ambulatory setting including blood tests, X-rays, and/or thoracic CT scans. If recurrence or metastasis (as defined below) was suspected, further clinical evaluations (e.g., bronchoscopical/ultra-sound/CT-guided transthoracic biopsy, follow-up PET-CT/MRI scan or skeletal scintigraphy) were performed according to the individual scenario. Information on overall survival (OS) was provided by the institutional statistical offices and calculated as the elapsed time between surgery and death or last follow-up in months. Information on disease-free survival (DFS) was achieved from the medical records and calculated as the time between surgery and clinical evidence of recurrent disease (defined as tumor recurrence in mediastinal/hilar lymph nodes/ipsilateral lung) or distant organ metastases in months.

### Surgically resected samples, immunohistochemistry and evaluation

FFPE blocks were cut into 4-µm-thick sections and stained with commercially available antibodies according to the manufacturer’s protocols for C-Myc (Abcam AB32072, dilution 1:100), L-Myc (Thermofisher PA5-41114, dilution 1:200), N-Myc (Cell Signaling D42BY, dilution 1:100), ASCL1 (BD Bioscience #555604, dilution 1:50), NEUROD1 (Abcam AB213725, dilution 1:100), YAP1 (Cell Signaling Technology #4912, dilution 1:200), and POU2F3 (Santa Cruz Biotechnology SC-293402, dilution 1:100). Briefly, after deparaffinization and incubation of the sections with 0.3% H_2_O_2_ for 30 min at room temperature, primary antibodies were applied on the tissue slides overnight at 4 °C. On the next day, tissue slides were incubated with secondary antibodies for 1 h at room temperature. Finally, DAB + Substrate Chromogen System (Dako, K3469) and counterstaining with hematoxylin was performed to visualize the staining results. Ultimately, all sections were scanned using the 3DHistech Midi Scanner System in order to provide digital evaluation by the Panoramic CaseViewer Software (Budapest, Hungary, Version 2.4.0.119028). Immunohistochemistry (IHC) results were independently evaluated and scored by two board-certified pathologists with high experience in pulmonary pathology. In case of relevant differences between the two ratings, the slide was re-evaluated. Notably, there are currently no standardized protocols for the evaluation of C-Myc, L-Myc, N-Myc, ASCL1, NEUROD1, YAP1, or POU2F3 expressions for surgically resected SCLC specimens. Therefore, we defined C-Myc, L-Myc, N-Myc, ASCL1, NEUROD1, YAP1, and POU2F3 expressions as positive if more than 1% of the tumor cells demonstrated prominent nucleocytoplasmic staining or as previously described [5].

### Statistical analysis

Continuous variables were presented as median, and in distinct cases (e.g., age of the study cohort), as range including minimum and maximum values. Categorical variables were presented as total numbers and frequencies, and the groups were compared by using the Chi-square test. If the expected frequency was < 5 in one group, the Fisher’s exact test was applied. Correlation analysis between categorical variables was achieved by using Pearson’s chi-squared and Cramér’s V approach.

For risk factor estimation, univariate Cox regression model analysis was applied and the outcomes were presented as hazard ratios (HR) including the corresponding 95% confidence intervals (CI). All risk factors with significant results on univariate analysis were included for multivariate analysis to be tested for robustness and independency. For survival curve estimation of OS and DFS, the Kaplan–Meier method was applied. For comparison of significant differences of Kaplan–Meier curves, the log-rank test was used. All tests were calculated in a two-sided manner and *p* values below 0.05 were considered statistically significant. In this study, all statistical and graphical illustrations were done by using GraphPad Prism 8 (GraphPad Software, Inc., USA) and SPSS Statistics Version 27.0 (IBM Corp., USA).

## Results

### Demographic description of the study cohort

**Fig. 1:**
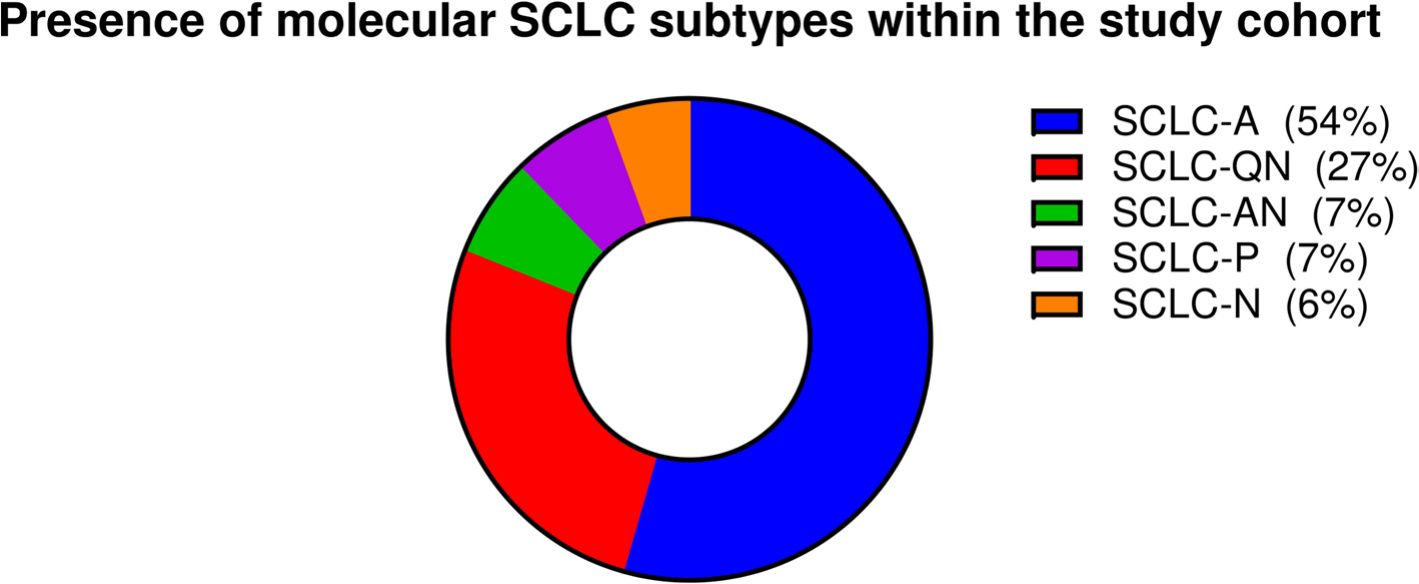
Frequency and distribution of major molecular SCLC subtypes within the study cohort after immunohistochemistry for ASCL1, NEUROD1, POU2F3, and YAP1. (SCLC-A: ASCL1-dominant subtype; SCLC-QN: quadruple-negative subtype; SCLC-AN: combined ASCL1/NEUROD1-dominant subtype; SCLC-P: POU2F3-dominant subtype; SCLC-N: NEUROD1-dominant subtype)

In total, 104 patients were included in the current study (Table 1). Median age of the patients at time of surgery was 64 (range 41–83) years. Fifty-four patients (52%) were male and 87 (83.7%) were former or current tobacco smokers. Hypertension (50.9%), chronic obstructive pulmonary disease (COPD, 41.3%), and diabetes mellitus (16.3%) were the most common comorbidities within the study cohort. Regarding the type of surgical resection, lobectomy (50.9%) was the most commonly performed procedure, followed by segmentectomy (13.5%), wedge resection (11.5%), and pneumonectomy (10.6%). According to the histopathological findings, 57 (54.8%) patients had limited-stage (= TNM stage I or II) SCLC, 44 (42.3%) patients had negative lymph nodes (= N0), and 42 (40.4%) individuals had T1 lesions. The majority of the study cohort (*n* = 63, 60.6%) received adjuvant treatment (*n* = 54 adjuvant chemotherapy without radiotherapy, *n* = 2 radiotherapy without chemotherapy, and *n* = 7 combined chemo-radiotherapy). All patients had an Eastern Cooperative Oncology Group (ECOG) performance status of 0 or 1. Ultimately, the most common molecular SCLC subtypes among the evaluated specimens was SCLC-A (ASCL1-dominant) with 54%, SCLC-QN (quadruple negative) with 27%, SCLC-AN (combined ASCL1/NEUROD1-dominant) and SCLC-P (POU2F3) with 7%, and SCLC-N (NEUROD1) with 6% of the cases (Fig. 1). Notably, no unique SCLC-Y (YAP1-dominant) subtype was identified.

**Table 1:** Clinicopathological characteristics of the study cohort. In total, 104 individuals undergoing surgical resection for SCLC were included in this study

	**Total (*n* = 104)**
**Age** (years, median, range)	64 (41–83)
< 65	56 (54%)
≥ 65	47 (45%)
N/A	1 (1%)
**Gender**	
Female	49 (47%)
Male	54 (52%)
N/A	1 (1%)
**Smoking status**	
Non-smoker	12 (11.5%)
Former or current smoker	87 (83.7%)
N/A	5 (4.8%)
**Most common comorbidities**	
Hypertension	53 (50.9%)
Chronic obstructive pulmonary disease (COPD)	43 (41.3%)
Diabetes mellitus	17 (16.3%)
**Surgery type**	
Wedge resection	12 (11.5%)
Segmentectomy	14 (13.5%)
Lobectomy	53 (50.9%)
Pneumonectomy	11 (10.6%)
Unspecified	14 (13.5%)
**Pathologic stage**	
I	39 (37.5%)
II	18 (17.3%)
≥ III	31 (29.8%)
N/A	16 (15.4%)
**Lymph node status**	
N0	44 (42.3%)
N1	23 (22.1%)
N2	17 (16.3%)
N/A	20 (19.2%)
**Tumor size**	
T1	42 (40.4%)
T2	22 (21.2%)
T3	11 (10.6%)
T4	12 (11.5%)
N/A	17 (16.3%)
**Adjuvant therapy** (chemotherapy and/or irradiation)	
Yes	63 (60.6%)
No	24 (23.1%)
N/A	17 (16.3%)
**Eastern Cooperative Oncology Group (ECOG) Performance Status 0–1**	104 (100%)

### Impact of clinicopathological characteristics on clinical outcome

**Fig. 2:**
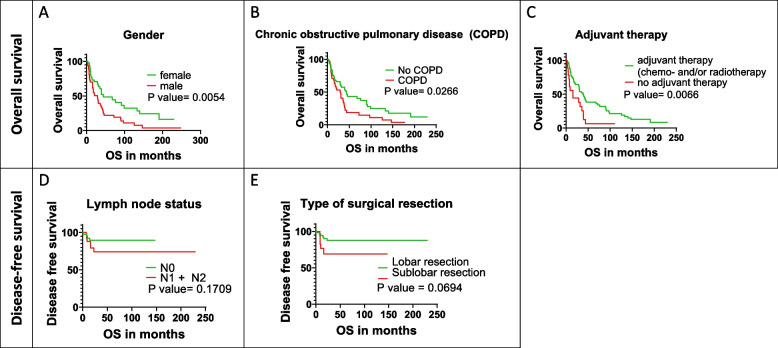
Clinicopathological features correlating with overall (OS) or disease-free survival (DFS) following surgery in SCLC patients. Men (**A**), patients with COPD (**B**), and those not receiving any type of adjuvant therapy (**C**) tended to have significantly worse OS. Patients with advanced lymph node stage (**D**) and those receiving sublobar resection (defined as wedge resection or segmentectomy (**E**), showed a statistically non-significant but clear tendency toward shorter DFS

Next, we aimed to determine whether distinct clinicopathological features of surgically treated SCLC patients associate with the postoperative outcome (Fig. 2). Male patients had significantly worse OS than female individuals (median OSs were 29 and 44 months, respectively, *p* = 0.0054). Similarly, patients with COPD had significantly shorter median OS (vs. non-COPD patients, 30 vs. 44 months, *p* = 0.0266). In contrast, patients receiving adjuvant treatment had a remarkably improved OS compared to patients undergoing surgery alone (40 vs. 16 months, *p* = 0.0066). Finally, none of the clinicopathological characteristics showed a statistically significant association with disease-free survival (DFS). However, patients with more advanced lymph node status (N1 or N2 vs. N0, 13 vs. 30 months, *p* = 0.1709) and patients receiving sublobar resection (i.e., wedge resection or anatomic segmentectomy) showed a tendency toward shorter DFS (compared to patients receiving lobectomy or pneumonectomy, 10 vs. 29 months, *p* = 0.0694).

### Expression patterns of Myc and their association with clinicopathological characteristics

**Fig. 3:**
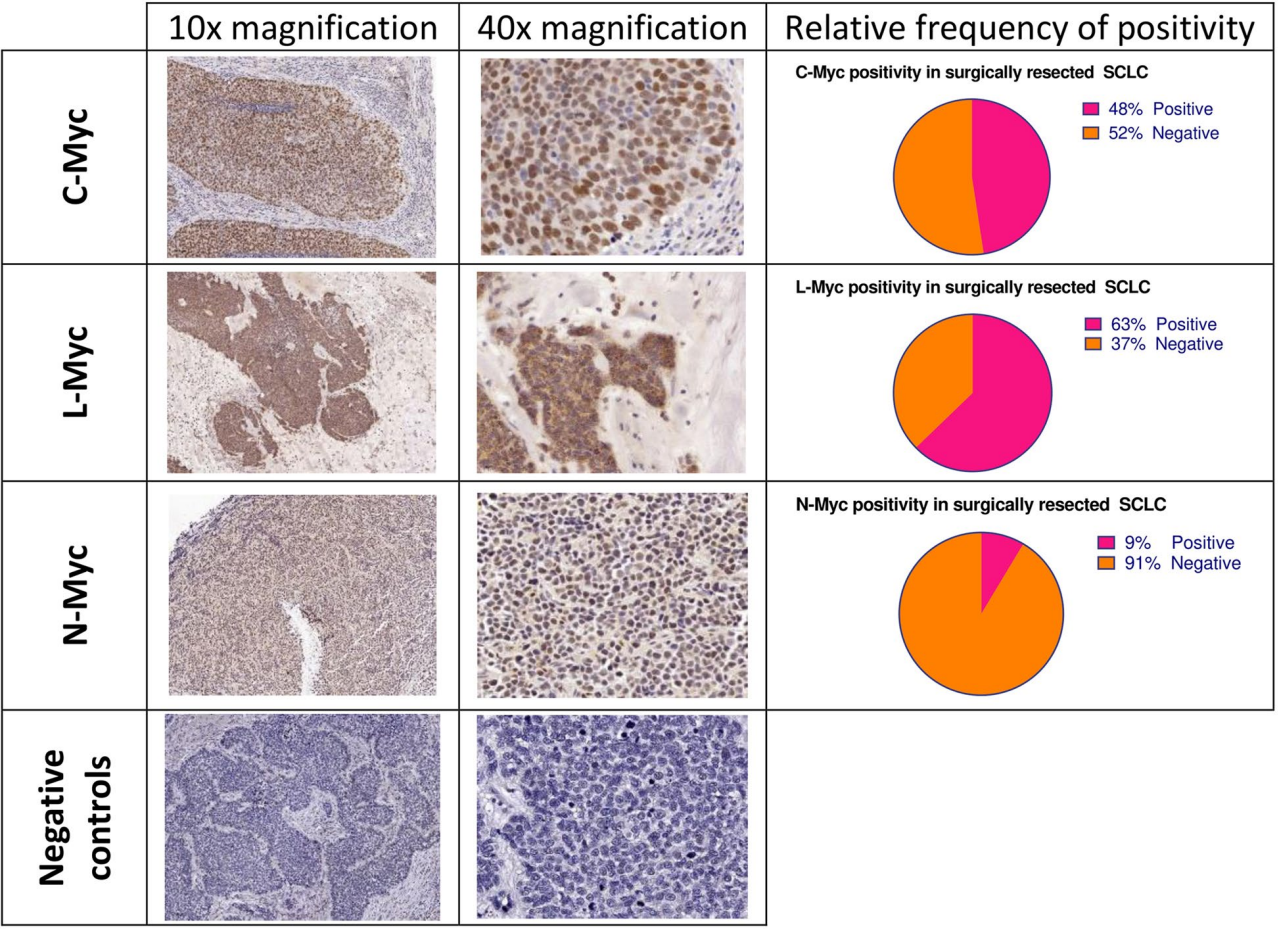
Representative images of C-Myc, L-Myc, and N-Myc expression detected by IHC in surgically resected SCLC specimens. The pie charts illustrate the relative frequencies of positivity concerning distinct MYC family members. Representative negative controls are shown at the bottom

Positive C-Myc expression by IHC was found in 48% of all cases (Fig. 3). L-Myc expression was even more pronounced present with positive rate of 63%. In contrast, N-Myc expression was clearly weaker since 9% of all specimens were classified as positive for N-Myc. Although none of the Myc family members were associated with distinct demographic features (Supplementary Table 1), N-Myc positivity significantly correlated with the SCLC-P (POU2F3 dominant) subtype (Pearson correlation: 0.6913, *p* = 0.0056, Table 2).

**Table 2:** Correlation between Myc family member positivity and major molecular SCLC subtypes within the study cohort

	**C-Myc positivity**		**L-Myc positivity**		**N-Myc positivity**	
	Pearson correlation	*p* value	Pearson correlation	*p* value	Pearson correlation	*p* value
**SCLC-A** (ASCL1-dominant subtype)	0.0889	0.6315	0.0752	0.7083	0.119	0.8231
**SCLC-QN** (Quadruple negative subtype)	0.0993	0.5777	0.0241	0.9203	0.1155	1
**SCLC-AN** (combined ASCL1/NEUROD1 dominant subtype)	0.1871	0.2029	0.174	0.2006	0.0979	1
**SCLC-P** (POU2F3-dominant subtype)	0.2239	0.0997	0.1857	0.2114	0.6913	0.0056*
**SCLC-N** (NEUROD1-dominant subtype)	0.1373	0.3368	0.0722	1	0.2336	0.2343

### Prognostic value of Myc and validation of independent predictors for clinical outcome

**Fig. 4:**
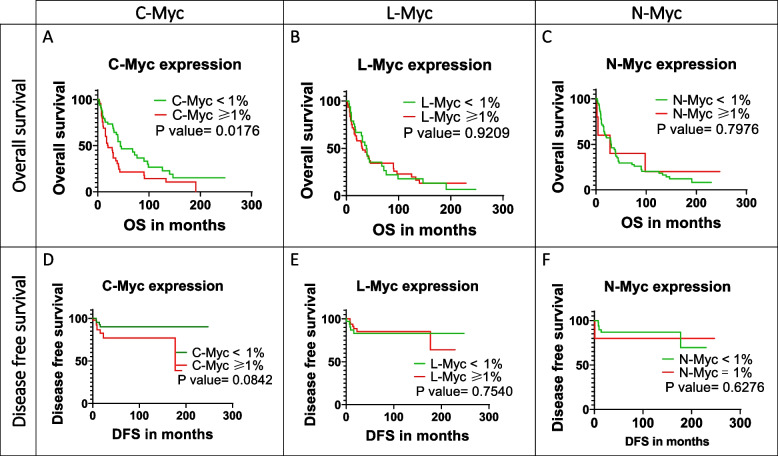
Kaplan–Meier survival curves for overall survival (**A**–**C**) and disease-free survival (**D**–**F**) according to the IHC protein expression status of MYC family members in surgically resected SCLC

Finally, we evaluated the prognostic value of Myc members in surgically resected SCLC patients. Regarding OS, positive C-Myc expression was significantly associated with worse overall outcome (20 months vs. 44 months compared to C-Myc negative patients, *p* = 0.0176, Fig. 4). Likewise, C-Myc positivity correlated with poor DFS (14 months vs. 32 months compared to C-Myc negative cases, *p* = 0.0842) by just nearly missing the level of statistical significance. In contrast, neither the expression of L-Myc or N-Myc demonstrated an association with OS or DFS.

**Fig. 5:**
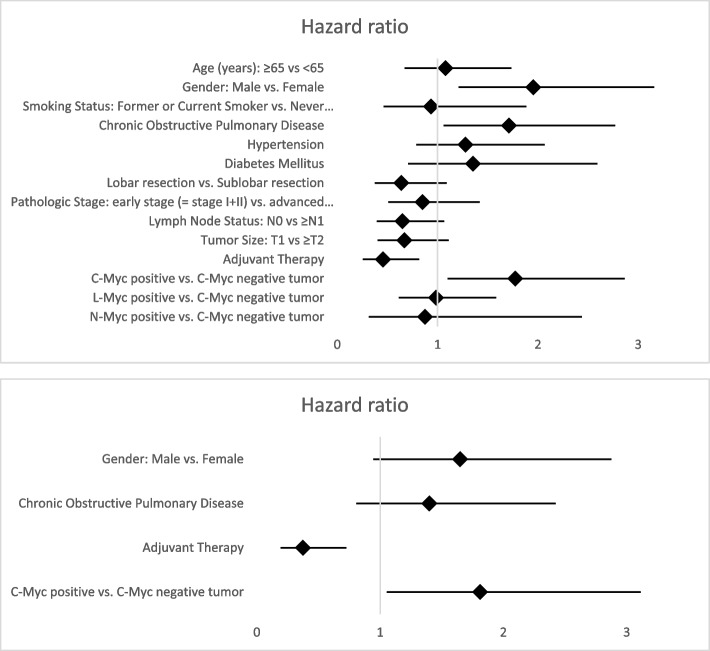
Forest map of univariate analysis for the evaluation of distinct clinicopathological features and differential Myc family members’ expression as predictors of overall survival (OS) following surgical resection in SCLC. Detailed results are included in Supplementary Table 2. Forest map of multivariate analysis for the evaluation of distinct clinicopathological features and differential Myc family members’ expression as predictors of overall survival (OS) following surgical resection in SCLC. Detailed results are included in Supplementary Table 2

**Fig. 6:**
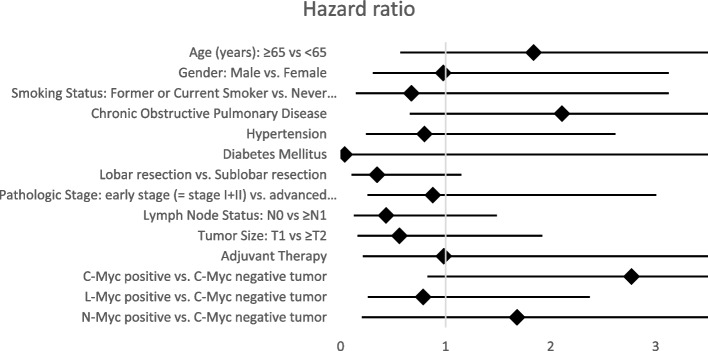
Univariate analysis for the evaluation of distinct clinicopathological factors and differential Myc family members’ expression status as predictors of disease-free survival (DFS) following surgical resection in SCLC. Detailed results are included in Supplementary Table 3

Ultimately, in order to test whether C-Myc positivity influences the survival outcomes independently of other clinicopathological variables, we performed univariate and multivariate analysis for all available characteristics for OS (Fig. 5 and Supplementary Table 2) and DFS (Fig. 6 and Supplementary Table 3). In univariate analysis, gender (male vs. female, hazard ratio (HR) = 1.954, confidence interval (CI) 95% 1.209–3.158, *p* = 0.006), COPD (HR 1.712, CI 95% 1.059–2.77, *p* = 0.028), and positive C-Myc expression (HR 1.774, CI 95% 1.099–2.865, *p* = 0.019) were clear risk factors for shorter OS while adjuvant treatment (HR 0.456, CI 95% 0.255–0.815, *p* = 0.008) represented a significant predictor of longer OS. In multivariate analysis, only positive C-Myc expression (HR 1.811, CI 95% 1.054–3.113, *p* = 0.032) and adjuvant treatment (HR 0.374, CI 95% 0.193–0.726, *p* = 0.004) remained reliable prognostic factors for OS.

Regarding DFS, univariate analysis did not demonstrate a statistically significant prognostic value for distinct clinicopathological factors or expression of any Myc member. Nevertheless, lobar resection (vs. sublobar resection, HR 0.348, CI 95% 0.106–1.148, *p* = 0.083) slightly missed the level of significance as a favorable predictor for DFS. In addition, univariate analysis also revealed a statistically non-significant but clearly increased risk for shorter DFS in case of C-Myc positivity (HR 2.770, CI 95% 0.829–9.259, *p* = 0.098).

## Discussion

In this study, we investigated the protein expression and prognostic impact of Myc family members in an international cohort of 104 surgically treated SCLC patients. C-Myc, L-Myc, and N-Myc showed heterogeneous expressions with the highest positivity rate for L-Myc, followed by C-Myc and N-Myc. In addition, positive C-Myc expression was identified as a novel prognostic biomarker robustly indicating impaired clinical outcome among surgically managed SCLC patients.

Therapeutic opportunities in various thoracic malignancies have gone through a remarkable improvement with continuously increasing OS rates over the past decades [23–25]. In contrast, only few therapeutic advancements with very modest survival benefits have been added to the treatment armamentarium in SCLC. Indeed, SCLC has remained one of the most disastrous thoracic malignancies with 5-year survival rates constantly plateauing below 10% since decades [26]. Although surgical resection combined with adjuvant platinum-based chemotherapy has been widely proved to offer acceptable long-term results, only a small number of SCLC patients can be treated with this approach [27, 28]. Unfortunately, the vast majority of SCLC patients already has advanced disease and metastasis at the time of initial diagnosis when local resection is not feasible anymore [29]. Inevitably, this results in a low number of surgically managed SCLC patients and thus a limited availability of surgically resected whole tissue specimens for research purposes. Regarding our study cohort, the overall number of a comparatively large group of 104 whole tissue (WTS) specimens of SCLC patients was achieved through a collaboration between two high-volume thoracic surgical departments. Also, we selected a study period of 20 years and decided to include specimens even from advanced stage SCLC patients to increase the total number of available specimens. Interestingly, we found remarkable associations between distinct clinicopathological characteristics and clinical outcomes. Male patients, patients with COPD and those not receiving an adjuvant treatment had clearly worse OS. Also, advanced lymph node stage and sublobar surgical resection showed a clear association with early recurrence. Our findings underline previous reports from comparable cohorts, demonstrating a clear impact of gender, lymph node status, lobar resection, and adjuvant treatment on clinical outcome [27, 30, 31]. Interestingly, the reason why females demonstrate a remarkably longer OS—irrespective of possible confounders such as age, stage, therapy, smoking history—in various entities including SCLC, remains a matter of ongoing debate [32]. One possible explanation has been linked to the protective effects of estrogen, which underlines the longer natural life expectancy of females both in humans and animals [33]. However, ongoing debates necessitate further elucidation for alternative explanations. Adjuvant chemotherapy should be mandatory for all surgically managed SCLC patients, yet we had a high proportion of cases not receiving adjuvant chemotherapy, which is similar to the findings in comparable cohorts [34, 35]. Additionally, nodal upstaging is a common phenomenon in surgically managed SCLC, which might have also resulted in a significant number of advanced-stage patients in our study cohort [36, 37]. With regards to the potentially increasing number of limited-stage lung cancer cases in nationwide lung screening programs and the ongoing debate to reinforce the role of surgery in current SCLC guidelines, these findings should endorse practicing clinicians to carefully perform preoperative staging, take individual clinicopathological factors into account, and consequently, manage all patients selected for surgery in a multidisciplinary manner to offer optimal long-term results [38–41].

For the evaluation of Myc expression in our surgically resected specimens, we decided to perform IHC as it is one the most well-established methods for protein expression determination and which has been previously proven to be a valuable approach for investigating Myc in cancerous tissue [42, 43]. Clearly, gene amplification rate might also be relevant when investigating the role of Myc, but there is already sufficient data showing a clear correlation between increased amplification of Myc and a poor outcome in various malignancies including SCLC [44–46]. Moreover, increased gene amplification rates of Myc have been previously shown to clearly correlate with increased Myc protein level and detectability by IHC [47]. Therefore, we focused on IHC and divided our cohort into completely negative samples versus samples showing at least 1% positivity. With this approach, we found that 63% of patients were positive for L-Myc followed by 48% for C-Myc and 9% for N-Myc. These rates are in line with previously reported tendencies toward higher L-Myc and C-Myc presence compared to a generally weaker N-Myc presence in SCLC [48]. Recent studies on mouse and human models demonstrate that Myc family members drive dynamic evolution of SCLC subtypes. In this context, MYC directly activates NOTCH signaling, thus reprogramming SCLC from ASCL1 + to NEUROD1 + and, finally, to non-neuroendocrine states [8, 9]. Moreover, in vivo and in vivo SCLC models have also shown direct associations between the abundance of distinct Myc family members and molecular SCLC subtypes (e.g., ASCL1/SCLC-A and MYCL/L-Myc or NEUROD1/SCLC-N and MYC/C-Myc) [4]. Although we did not identify clear relations between positivity for Myc and clinicopathological characteristics, there was weak evidence that patients with N-Myc positivity were more likely to have a POU2F3-dominant molecular SCLC subtype. However, considering the low total number of N-Myc positive and POU2F3 SCLC cases in our cohort, this finding should be interpreted with caution. Altogether, because surgery is rarely performed in SCLC and thus only a few studies consisting of small cohorts provided controversial data on the expression and prognostic role of Myc family members in surgically managed SCLC patients, caution must be taken when interpreting our findings [49, 50]. Of note, Qin et al. analyzed the expression levels of C-Myc and L-Myc in *n* = 46 resected SCLC cases by IHC. Notably, they used deviant definitions for positive staining results (in case of C-Myc: ≥ 40% positive cells, in case of L-Myc: ≥ 10% positive cells) and found an overall low prevalence of C-Myc (4/46) and L-Myc positivity. Moreover, in their study, patients with positive C-Myc and L-Myc expression had a tendency toward a favorable clinical outcome. In contrast, another recent study analyzed C-Myc, L-Myc, and N-Myc expression levels in *n* = 83 surgically resected high-grade neuroendocrine carcinoma (HGNEC) lung specimens, of whom *n* = 36 specimens were SCLC cases [50]. In this study, specimens were defined as positive if ≥ 10% of the cells showed a positive staining for C-Myc, L-Myc, or N-Myc. Ultimately, comparable to the findings of our study, C-Myc positivity was found to be an independent prognostic factor indicating unfavorable clinical outcome. Clearly, further studies are warranted to evaluate the prognostic value of C-Myc in surgically resected SCLC patients.

The prognostic and predictive relevance of molecular SCLC subtypes has been just recently evaluated by our group in a large-scaled multicentric study including *n* = 386 surgically resected specimens and 26 human cell lines [5]. In that study, high ASCL1 expression was an independent negative prognosticator in surgically treated SCLC patients. In contrast, in a univariate model, high POU2F3 expression was associated with improved survival outcomes. In addition, we found remarkable associations between distinct molecular SCLC subtypes and the efficacy of standard-of-care and targeted therapeutics in vitro [5]. Notably, most patients in the current study had SCLC-A tumors, and no distinct YAP1-defined subtype could be distinguished. This is in line with our previous findings. Indeed, the results of other protein-based studies are also questioning the subtype-defining potential of YAP1 and arguing in favor of a unique subtype showing negative staining with the subtype markers ASCL1, NEUROD1, and POU2F3 [5, 51]. However, since the patient pool of the current study (all 104 cases) overlapped with our multicenter research [5], we did not aim to republish our previous findings on the prognostic role of molecular SCLC subtypes in the smaller current dataset.

Regarding their prognostic impact, L-Myc and N-Myc did not demonstrate any correlation with the clinical outcome in our surgical SCLC cohort. However, patients with C-Myc positive tumors had significantly impaired survival outcomes and also a clearly increased risk for shorter OS according to multivariate Cox proportional analysis. These findings might be explicable by the solid preclinical evidence demonstrating that Myc plays a key role in SCLC disease progression [8, 9]. Accordingly, tumors with higher C-Myc presence may have a more aggressive nature and result in accelerated progression, resulting in early recurrence and shorter OS. In line with our findings, there are numerous methodically comparable studies reporting on similar results in further thoracic and extra-thoracic malignancies [14, 52, 53]. In addition, preclinical evidence highlights a potential link between increased C-Myc activity and acquired resistance to standard-of-care chemotherapeutics, which might serve as a potential explanation why SCLC patients with a higher C-Myc abundance face a dismal overall prognosis [12, 54]. Altogether, Myc represents a promising targetable molecule in SCLC. Indeed, preclinical SCLC models clearly demonstrated efficacy of indirect or direct inhibitory strategies for Myc [20, 21, 55]. Of note, the first clinical trials including patients with various solid tumor types have recently been started to evaluate the therapeutic potency of direct Myc inhibition with OMO-103 [19, 56]. Altogether, clinical evaluation of Myc targeting in SCLC is highly warranted. We believe that our results on the prognostic role of C-Myc positivity in surgically treated patients are hypothesis-generating in their current form. However, in order to consider C-Myc positivity as a novel biomarker for the selecting eligible SCLC patients for surgical resection, further validation is needed. Nevertheless, C-Myc expression can still be considered a prognostic marker that can aid in defining the optimal follow-up strategy for patients who underwent surgical resection.

Our study has certain limitations that must be alluded. First, although we collected a comparatively large cohort of surgically treated SCLC cases, the overall number of included patients still remained relatively small. Second, due to the retrospective study design, the extent of information on clinicopathological and follow-up data was limited. Third, as there are no standardized protocols for the evaluation of Myc or the subtype-specific SCLC marker expressions by IHC, reproducibility of our results might be weakened when applying differing approaches of evaluation. Fourth, the current study solely included FFPE samples and thus Myc expressions may significantly differ in fresh-frozen samples [57]. Finally, although they could have provided valuable information on the role of these molecules in surgically managed SCLC patients, genomic data on MYC, MYCL, MYCN, ASCL1, NEUROD1, POU2F3, and YAP1 expressions were not available in our cohort.

## Conclusion

Protein expression pattern of C-Myc, L-Myc, N-Myc, and their correlation with molecular SCLC subtypes is heterogeneous in surgically resected SCLC patients. In addition, C-Myc positivity is a robust prognosticator of impaired OS among surgically managed SCLC patients. More studies are warranted to investigate the role and clinical implications of Myc family members and subtype-specific markers in SCLC.

### Supplementary Information

**Additional file 1: Supplementary Table 1.** Correlation of Myc family members with clinicopathological characteristics of the study population (*n* = 104).

**Additional file 2: Supplementary Table 2.** Univariate and multivariate analysis for the evaluation of distinct clinicopathological features and differential Myc family members expression as predictors of overall survival (OS) following surgical resection in SCLC.

**Additional file 3: Supplementary Table 3.** Univariate and multivariate analyses for the evaluation of distinct clinicopathological factors and differential Myc family members’ expression status as predictors of disease-free survival (DFS) following surgical resection in SCLC.

## Data Availability

Data were generated by the authors and available on reasonable request.

## Abbreviations

CI: Confidence intervals
DFS: Disease-free survival
FFPE: Formalin-fixed paraffin-embedded
HR: Hazard ratios
IHC: Immunohistochemistry
NCCN: National Comprehensive Cancer Network
OS: Overall survival
SCLC: Small cell lung cancer
SCLC-A: ASCL1-dominant subtype
SCLC-AN: Combined ASCL1/NEUROD1-dominant subtype
SCLC-P: POU2F3 dominant subtype
SCLC-N: NEUROD1-dominant subtype
SCLC-QN: Quadruple negative subtype
SCLC-Y: YAP1-dominant subtype
WTS: Whole tissue specimens
