# Supplementary material for: C-Myc protein expression indicates unfavorable clinical outcome in surgically resected small cell lung cancer
Source: World J Surg Oncol. 2024 Feb 19;22:57. doi: 10.1186/s12957-024-03315-7 (PMC10875875; doi:10.1186/s12957-024-03315-7)
Supplement: Supplementary file 1 — Additional file 1: Supplementary Table 1. Correlation of Myc family members with clinicopathological characteristics of the study population (n = 104). [file 12957_2024_3315_MOESM1_ESM.docx]

**Supplementary Table 1.** Correlation of Myc family members with clinicopathological characteristics of the study population (n=104).

|  | **C-Myc Positivity** | | **L-Myc Positivity** | | **N-Myc Positivity** | |
| --- | --- | --- | --- | --- | --- | --- |
|  | Pearson correlation | p-value | Pearson correlation | p-value | Pearson correlation | p-value |
| **Age**  (<65 vs. >= 65 years) | 0.0741 | 0.5839 | 0.0689 | 0.6242 | 0.0239 | 0.8065 |
| **Gender**  (female vs. male) | 0.1205 | 0.3102 | 0.0141 | 0.9203 | 0.1871 | 0.256 |
| **Smoking Status** (non-smoker vs. former- or current smoker) | 0.0226 | 0.9203 | 0.1563 | 0.2222 | 0.1029 | 1 |
| **Hypertension** (yes vs. no) | 0.1474 | 0.2059 | 0.0352 | 0.8875 | 0.1036 | 0.7401 |
| **Chronic Obstructive Pulmonary Disease**  (yes vs. no) | 0.1837 | 0.1042 | 0.0202 | 1 | 0.0686 | 1 |
| **Diabetes Mellitus** (yes vs. no) | 0.102 | 0.4543 | 0.0319 | 1 | 0.0866 | 1 |
| **Pathological Stage** (stage I+II vs. stage ≥III) | 0.1678 | 0.1809 | 0.0264 | 1 | 0.1114 | 0.7773 |
| **Lymph Node Status** (N0 vs. ≥N1) | 0.0269 | 1 | 0.0841 | 0.5902 | 0.0189 | 0.7083 |
| **Tumor Size** (T1 vs. ≥ T2) | 0.0285 | 1 | 0.0695 | 0.6714 | 0.1083 | 0.7642 |
| **Adjuvant Chemotherapy**  (yes vs. no) | 0.1278 | 0.3865 | 0.039 | 0.729 | 0.0348 | 0.5598 |
