# Supplementary material for: C-Myc protein expression indicates unfavorable clinical outcome in surgically resected small cell lung cancer
Source: World J Surg Oncol. 2024 Feb 19;22:57. doi: 10.1186/s12957-024-03315-7 (PMC10875875; doi:10.1186/s12957-024-03315-7)
Supplement: Supplementary file 2 — Additional file 2: Supplementary Table 2. Univariate and multivariate analysis for the evaluation of distinct clinicopathological features and differential Myc family members expression as predictors of overall survival (OS) following surgical resection in SCLC. [file 12957_2024_3315_MOESM2_ESM.docx]

**Supplementary Table 2.**

Univariate and multivariate analysis for the evaluation of distinct clinicopathological features and differential Myc family members expression as predictors of overall survival (OS) following surgical resection in SCLC.

| Predictors of OS | | Univariate analysis | | | Multivariate analysis | | |
| --- | --- | --- | --- | --- | --- | --- | --- |
|  |  | *HR* | *CI 95%* | *P value* | *HR* | *CI 95%* | *P value* |
| Clinicopathological factors |  | | | | | | |
|  | Age (years): ≥65 vs <65 | 1.079 | 0.671-1.735 | 0.755 |  |  |  |
|  | Gender: Male vs. Female | 1.954 | 1.209-3.158 | 0.006* | 1.648 | 0.945-2.875 | 0.078 |
|  | Smoking Status:  Former or Current Smoker vs. Never Smoker | 0.934 | 0.463-1.885 | 0.848 |  |  |  |
|  | Chronic Obstructive Pulmonary Disease | 1.712 | 1.059-2.77 | 0.028* | 1.399 | 0.807-2.423 | 0.232 |
|  | Hypertension | 1.277 | 0.789-2.066 | 0.320 |  |  |  |
|  | Diabetes Mellitus | 1.353 | 0.706-2.593 | 0.362 |  |  |  |
|  | Lobar resection vs. Sublobar resection | 0.638 | 0.374-1.09 | 0.100 |  |  |  |
|  | Pathologic Stage: early stage (= stage I+II) vs. advanced stage (≥ stage III) | 0.849 | 0.509-1.418 | 0.532 |  |  |  |
|  | Lymph Node Status:  N0 vs ≥N1 | 0.649 | 0.395-1.065 | 0.087 |  |  |  |
|  | Tumor Size: T1 vs ≥T2 | 0.669 | 0.403-1.109 | 0.119 |  |  |  |
|  | **Adjuvant Therapy** | **0.456** | **0.255-0.815** | **0.008*** | **0.374** | **0.193-0.726** | **0.004*** |
| Myc family members | **C-Myc positive vs. C-Myc negative tumor** | **1.774** | **1.099-2.865** | **0.019*** | **1.811** | **1.054-3.113** | **0.032*** |
|  | L-Myc positive vs. C-Myc negative tumor | 0.985 | 0.613-1.583 | 0.949 |  |  |  |
|  | N-Myc positive vs. C-Myc negative tumor | 0.875 | 0.314-2.438 | 0.798 |  |  |  |
