# Supplementary material for: C-Myc protein expression indicates unfavorable clinical outcome in surgically resected small cell lung cancer
Source: World J Surg Oncol. 2024 Feb 19;22:57. doi: 10.1186/s12957-024-03315-7 (PMC10875875; doi:10.1186/s12957-024-03315-7)
Supplement: Supplementary file 3 — Additional file 3: Supplementary Table 3. Univariate and multivariate analyses for the evaluation of distinct clinicopathological factors and differential Myc family members’ expression status as predictors of disease-free survival (DFS) following surgical resection in SCLC. [file 12957_2024_3315_MOESM3_ESM.docx]

**Supplementary Table 3.**

Univariate and multivariate analyses for the evaluation of distinct clinicopathological factors and differential Myc family members’ expression status as predictors of disease-free survival (DFS) following surgical resection in SCLC.

| Predictors of DFS | | Univariate analysis | | |
| --- | --- | --- | --- | --- |
|  |  | *HR* | *CI 95%* | *P value* |
| Clinicopathological factors |  |  |  |  |
|  | Age (years): ≥65 vs <65 | 1.837 | 0.569-5.924 | 0.309 |
|  | Gender: Male vs. Female | 0.977 | 0.306-3.124 | 0.969 |
|  | Former or Current Smoker vs. Never Smoker | 0.674 | 0.146-3.124 | 0.614 |
|  | Chronic Obstructive Pulmonary Disease | 2.108 | 0.660-6.733 | 0.208 |
|  | Hypertension | 0.798 | 0.244-2.617 | 0.710 |
|  | Diabetes Mellitus | 0.039 | 0.000-58.636 | 0.385 |
|  | Lobar resection vs. Sublobar resection | 0.348 | 0.106-1.148 | 0.083 |
|  | Pathologic Stage: early stage (= stage I+II) vs advanced stage (≥ stage III) | 0.878 | 0.257-3.004 | 0.836 |
|  | Lymph Node Status:  N0 vs ≥N1 | 0.433 | 0.126-1.488 | 0.184 |
|  | Tumor Size: T1 vs ≥T2 | 0.560 | 0.163-1.920 | 0.356 |
|  | Adjuvant Therapy | 0.982 | 0.212-4.547 | 0.981 |
| Myc family members | C-Myc positive vs. C-Myc negative tumor | 2.770 | 0.829-9.259 | 0.098 |
|  | L-Myc positive vs. C-Myc negative tumor | 0.786 | 0.260-2.372 | 0.669 |
|  | N-Myc positive vs. C-Myc negative tumor | 1.680 | 0.202-13.986 | 0.631 |
